# A Tool for Sheep Product Quality: Custom Microarrays from Public Databases 

**DOI:** 10.3390/nu1020235

**Published:** 2009-12-04

**Authors:** Silvia Bongiorni, Giovanni Chillemi, Gianluca Prosperini, Susana Bueno, Alessio Valentini, Lorraine Pariset

**Affiliations:** 1 Dipartimento di Produzioni Animali, Università della Tuscia, 01100, Viterbo, Italy; Email: alessio@unitus.it (A.V.); pariset@unitus.it (L.P.); 2 Consorzio per le Applicazioni di Supercalcolo Per Università e Ricerca, 00185, Roma, Italy; Email: g.chillemi@caspur.it (G.C.); g.prosperini@caspur.it (G.P.); Susana.Bueno@caspur.it (S.B.)

**Keywords:** nutrigenomics, microarray, dairy products, mammary gland, milk quality, sheep, Sarda, Gentile di Puglia

## Abstract

Milk and dairy products are an essential food and an economic resource in many countries. Milk component synthesis and secretion by the mammary gland involve expression of a large number of genes whose nutritional regulation remains poorly defined. The purpose of this study was to gain an understanding of the genomic influence on milk quality and synthesis by comparing two sheep breeds with different milking attitude (Sarda and Gentile di Puglia) using sheep-specific microarray technology. From sheep ESTs deposited at NCBI, we have generated the first annotated microarray developed for sheep with a coverage of most of the genome.

## 1. Introduction

Milk and dairy products are an essential food and economic resource in many countries. Milk provides the primary source of nourishment for mammals’ offspring before their adult diet and contains the principal nutrients plus a huge number of micronutrient molecules, some of them with still unknown properties [1,2]. Therefore, the quality of milk and its control is becoming increasingly important. Milk component synthesis and secretion by the mammary gland varies dramatically across species and involves the expression of a large number of genes whose nutritional regulation remains poorly defined [3]. *Nutritional genomics* is an integrated science which studies gene expression to identify genetic and nutritional effects of a diet (the nutrient influence) on a single individual; while *nutrigenetics* seeks to understand the individual genetic differences which affect response to diet [4]. Knowledge of mammary uptake of nutrients, biosynthesis pathways, and the relation between diet and milk composition have been achieved in many studies [5,6,7,8]. Although much is known about the biochemistry of milk synthesis, the regulatory and cellular signaling systems of mammary gland are not well understood [9]. In dairy animals, mammary gland undergoes huge functional and metabolic adaptation to prepare lactogenesis. In all mammals, lactogenesis is characterized by two stages [10,11,12,13]. During the first stage (stage 01), which starts few weeks before parturition, the mammary gland differentiates for secreting colostrum and milk proteins. After parturition (stage 02), the metabolic activity increases the levels of milk production. Milk yield significantly rises during the first few weeks of lactation. During this period a well-studied set of genes, involved in milk synthesis, also increases its expression [12,14,15,16]. After the lactation peak, milk synthesis and qualified gene expression gradually decrease [14,15]. The end of milking activates the involution of the mammary gland which is characterized by epithelial cell death and by the mammary adipose tissue remodeling [17,18]. In dairy animals the nonlacting period, commonly referred to as the dry period between two lactations, is very important for milk production. A dry period of 40–60 days is necessary for optimal milk production during the next lactation [19].

In Italy, sheep is the second species in economic importance as a milk supply. Milk yield and composition, as well as lactation length, can fluctuate between breeds and within breeds. In normal sheep milk, fat ranges from 6% to 9%, protein from 4% to 7%, total solids from 17% to 21% and lactose from 4% to 6% [20]. Also other milk components implicated in human health vary considerably among breeds. Recently, Signorelli and collaborators [21] analyzed milk quality parameters and milk fatty acid profiles of three Italian breeds, Altamurana, Gentile di Puglia and Sarda, finding significant differences between breeds. The lowest content of saturated fatty acids (SFAs) was estimated in Gentile di Puglia breed, while mono-unsaturated FAs (MUFAs) were lowest in the Altamurana. No differences between breeds were found for conjugated linoleic acid (CLA) and poly-unsaturated FAs (PUFAs). Cheese quality is expected to be influenced by the differences between breeds in milk fatty acid contents [21].

The comparative analysis of some sheep breeds with different attitude to milk production could demonstrate the association between genetic variants and milk quality [21]. Candidate genes responsible for milk composition were intensively analyzed by Moioli and collaborators [22] to identify the molecular mechanisms underlying sheep milk quality. Among milk protein genes, the major effects were assessed for the αs1-casein, k-casein, β-lactoglobulin. Other important genes are those implicated in fatty acid metabolism, such as ACACA, SCD, LPL and DGAT1 [22]. However, in order to improve the overall picture, many more genes need to be deeply investigated.

Microarray technology is a powerful tool that helps to explore an organism transcriptome by measuring, in a particular cell or tissue, the expression levels of thousands of genes simultaneously. In livestock species, the microarray technology was discussed and reviewed as potential nutrigenomics tools, in the context of its economic benefits and improvement of food quality and safety in dairy and meat industries [23,24,25]. However, microarrays have been designed for very few livestock species. Moreover, the few devices so far developed, feature a largely incomplete coverage of the genome [26].

The objective of this study was to evaluate temporal changes in mammary gene network expression profiles by comparing two sheep breeds with different milking attitude. Gentile di Puglia (or Merino di Puglia, Pugliese Migliorata, Merino d'Italia, Merino Gentile) is a fine wooled breed from southern Italy. Development of this breed began in the 15th century, but the primary improvement began from the 18th century onwards. The breed was developed by crossing Spanish Merino with the local breeds. Today the selection objective of Gentile is focused onto meat production. Sarda is an Italian breed with high usefullness in milk production. It is widespread, mostly in Sardinia and in Central Italy, and representing 40% of the Italian ovine population.

In this study, we used a sheep-specific microarray chip technology covering most of the species’ transcriptome, representing the first annotated microarray developed for sheep with a covering of 50% of the genome [26]. The chip was generated from sheep ESTs deposited at NCBI and carries 21,743 non-redundant features in quadruplicate, 73.4% of which are fully annotated and corresponding to 10,190 genes. We analyzed the mammary transcriptome using biopsies from individuals of Gentile di Puglia and Sarda at two lactation stages to assess the differences between breeds, with the aim to identify genes controlling milk composition and their metabolic pathways.

## 2. Results and Discussion

We succefully hybridized eight microarray slides (four slides per lactation stage). Since every spot was replicated four times, for each lactation stage we performed 16 gene replicates (see Table 1).

In wet lab experiments 213 genes resulted differentially expressed between the two breeds at stages 01 (Table 2, Appendix) and 36 genes at stage 02 (Table 3, Appendix), with |FC| > 1.3, and p-value ≤ 0.05. The patterns of differentially expressed genes in *Ovis aries* were fully reproducible (see experimental section). At stage 01, 70 genes are upregulated in Gentile while 143 are upregulated in Sarda (Table 2, Appendix). At stage 02, only 8 genes are upregulated in Gentile while 28 are upregulated in Sarda, which is by far the most productive dairy breed (Table 3, Appendix).

**Figure 1:**
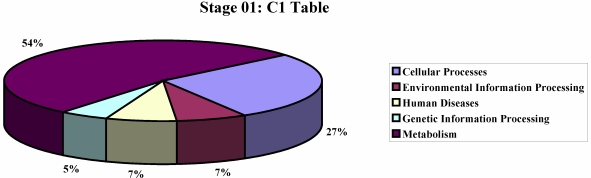
KEGG pathway analysis of the differentially expressed genes at stage 01.

**Figure 2:**
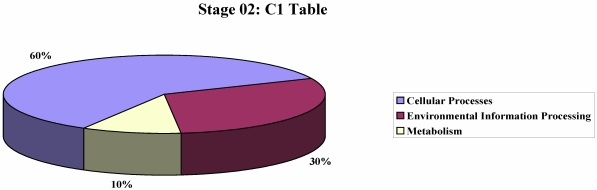
KEGG pathway analysis of the differentially expressed genes at stage 02.

We performed an analysis to show the most represented KEGG pathways among the differentially expressed genes (Figure 1, Figure 2), in order to identify molecular differences in milk synthesis between breeds and to identify genes controlling milk production and correlated metabolic pathways.

At stage 01, among the 143 upregulated genes in Sarda, we could recognize caseins αS2, β and K. In addition to milk protein genes, we identified genes involved in processes linked to both lactation and mammary involution, as oxidative metabolism, apoptosis, cell cycle control, oncogenes, ubiquitination pathway and cell communication (focal adhesion adherens junction), endocrine system (insulin signaling pathway, adipocytokine signaling pathway) [27]. The KEGG pathways (like amino acid and carbohydrate metabolism, glycan biosynthesis, cell communication, cell growth and death and the immune system) were significantly (p < 0.05) enriched. The molecular events underlying mammary development during pregnancy, lactation and involution are incompletely understood. The processes of lactation include the development of mammary tissue, as well as the synthesis and secretion of milk. After parturition, the proliferation and differentiation of mammary secretory cells lead to an increase in milk secretion, whereas after lactation peak, milk production declines largely because of apoptotic mammary cell death, which exceeds cell proliferation. The development of mammary gland is spatially regulated by the communication of the mammary epithelium with the extracellular matrix (ECM) through a family of adhesion receptors called integrins. Integrins, in response to both hormones and growth factors, support cells in proliferation, accurate morphological organisation, as well as in milk secretion. Cell adhesion to the ECM plays a key role in alveolar survival, morphogenesis and function [28]. In this context, we could observe a significant difference, between the two breeds, in expression of genes involved in extracellular matrix formation and cell adhesion (TJP1 upregulated in Gentile, CDH5 and TNXB upregulated in Sarda). Remarkably, during stage 01, the expression of the oncogene VAV3 is higher in Gentile, while one of the initiators of apoptosis CFLAR (CASP8 and FADD-like apoptosis regulator) increases in Sarda. Apoptosis, in fact, occurs during involution of mammary gland in cattle [29,30], and an overexpression of many apoptosis-related genes during lactation was recently reported [31]. At stage 01 (early lactation), we found a differential expression of genes, like USP9X, involved in the ubiquitination pathway in Sarda. The protein ubiquitination pathway is the most significantly enriched pathway during both lactation and involution [32]. Another category of genes found differentially expressed between the two breeds encompasses genes involved in oxidoreductase activity, like cytochrome C oxidase, NADH dehydrogenase and ferritin. The activity of cytochrome C oxidase was found to increase from late pregnancy to the first days of lactation [33,34]. The overall expansion of oxidative metabolism is a response to the increased energy demands of the lactation period. At stage 01 we observed an upregulation of cytochrome C oxidase, NADH dehydrogenase and ferritin in Gentile di Puglia. This may reflect the different lactation persistence, which is lower in Gentile di Puglia (60–150 days) as compared to Sarda (210 days). 

At stage 02, some interesting genes as those encoding casein K, and proteins involved in oxidoreductase activity (like TGOLN2 and FTH1) and in ECM-interaction (like COL1A2), resulted overexpressed in Sarda. Finally, we can observe in Sarda an overexpression of genes implicated in lipolysis, like lipase (DAGLB) and phospholipase (PLD3). Several studies on different kind of cheeses have demonstrated that the fatty acid (FA) profile of raw milk influences cheese characteristics [22]. Lipolysis is particularly important in sheep cheeses due to the high fat content and lipase activity [35]. In this perspective, we like to stress that in the World the main output of sheep husbandry is cheese making.

**Figure 3:**
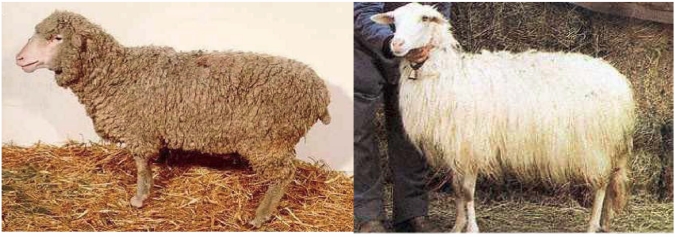
The two breeds analyzed: Gentile di Puglia (left) and Sarda (right).

## 3. Experimental Section

### 3.1. Animals and Sampling

Whole mammary gland tissue samples were collected from four lactating individuals of two sheep (*Ovis aries*) breeds, Gentile di Puglia and Sarda (Figure 3). Lactating mammary tissue were taken at two lactation stages (first record, stage 01: 6 days after lambing; second record, stage 02: 44 days after lambing) in both breeds. Tissues from mammary gland were immersed in RNA*later* (Sigma) and stored at −20 °C.

### 3.2. RNA Extraction

Tissues were subjected to RNA extraction with ice-cold TRIzol (Invitrogen) and using RNeasy Midi Kit columns (Qiagen). RNA integrity was assessed by electrophoretic analysis of 28S and 18S rRNA subunits. The purity of RNA and preliminary concentration were assessed with a spectrophotometer (GeneQuant*pro*). A260/A280 ratio was >1.9. 

### 3.3. RNA Amplification and Labeling

RNA was quantified using a DTX fluorimeter (Beckman Coulter) using the Quant-iT kit (Invitrogen). Aliquots of 1 μg were amplified and Cy3/Cy5 labeled using the Kreatech Diagnostics kit.

### 3.4. Microarray Study Design and Hybridization

We designed an oligonucleotide chip from sheep ESTs deposited at NCBI. The oligonucleotide microarray platform is electrochemically synthesized and contains 21,743 non-redundant features in quadruplicate, 73.4% of which are fully annotated corresponding to 10,190 genes. A genome assembly for *Ovis aries* is not yet available, but considering the number of genes in the bovine genome (22,000), we estimate to have a 50% coverage of the sheep genome. We achieved very good technical outcomes, as reproducible patterns of differentially expressed genes (in each slide, replicates show a coefficient of variation <0.25 for differentially expressed genes with P < 0.01) [26]. Oligos were generated *in situ* on the chip using the Combimatrix (Seattle, WA, USA) equipment. Platform and microarray data have been deposited in the NCBI GEO database (Platform Accession GPL9461; Series Accession GSE18619). 

The labeled aRNA was fragmented into 35-200-base fragments and then hybridized onto the slide according to Combimatrix’s instructions. Hybridization was performed overnight at 50 °C. After hybridization, arrays were washed and scanned with a ScanArray Lite (Perkin Elmer) laser scanner. Microarray Imager 5.9.3 software was used to extract feature data from microarray fluorescence images.

The microarray study was designed as described in Table 1. Separate microarrays were used for individual samples. At each stage, RNA of from one lactating individual of each breed was labeled in dual color using Cy3 and Cy5 fluorochromes. RNA aliquots from the same stage of distinct breeds, labeled with different fluorocromes, were hybridized on the same microarray slide. We performed two technical microarray replicates per stage (four slides). The entire microarray experiment was repeated starting from a new RNA extraction of the same tissue sample (see Table 1), for a total of eight slides.

**Table 1:** Microarray experimental design.

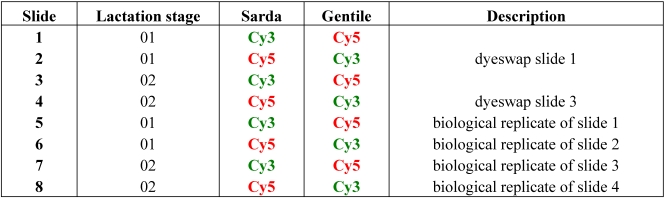

### 3.5. Microarray Data Analysis

#### Single microarray step

Saturated (foreground median intensity, FMI, over the limits) and bad spots were flagged using the software Microarray Imager 5.9.3 (CombiMatrix). For each channel (Cy5, red and Cy3, green) the mean of empty spots FMI (E) was calculated. Non-empty spots with FMI to E ratio below 1.5 at least in one channel were filtered, together with flagged spots. We calculated the M and the A value (M = log_2_(R/G), A = (log_2_(R*G))/2, R = red FMI, G = green FMI) for each spot in order to obtain a measure of the differential expression for the two conditions analysed (M) and the log-mean of the spot intensity (A).

#### Paired dye-swap microarrays step and normalization

Systematic bias in the data was removed by applying the dye-swap normalization, that makes use of the reverse labelling in the two microarray replicates in order to remove the intrinsic difference of the two fluorochromes output [36]. In particular we paired each microarray with its dye-swap by calculating a new M value, M_D_ = (M_1_ − M_2_)/2, where M_1_ is the M value for an experiment and M_2_ is the M value calculated for the correspondent experiment with inverse fluorochromes, and a new A value, A_D_ = A_1_ + A_2_)/2, where A_1_ is the A value for an experiment and and A_2_ is the A value calculated for the correspondent experiment with inverse fluorochromes. In order to remove intensity based bias we also applied the lowess normalization [37], obtaining a new M value (M_L_) from M_D_.

#### Significance analysis

For each lactation stage, we performed one sample t-test to establish, for each gene, if the mean of the M_L_ values (uM_L_) was significantly different from 0, and corrected the p-value for multiple comparisons with the Benjamini and Hochberg False Discovery Rate [38]. Finally, only genes with a satisfactory effect (absolute value of the fold change |FC*| > 1.3, FC = 2^ uM_L_, if FC<0, FC* = −1/FC, if FC > 0, FC* = FC) and a significant p-values were considered. The statistical significance of the enrichment for the KEGG pathways of interest was computed using the hypergeometric test [39].

## 4. Conclusions

Sheep farming is very important for cheese production and the fatty acid and protein composition of raw milk is crucial in the cheese making process. The fatty acid profile of raw milk has been demonstrated to affect cheese characteristics and differentiate new types of cheese [21,40]. The genetic differences between breeds on milk quality are likely to affect also cheese quality and could be a marker to carry out genetic improvement plans of local and endangered sheep breeds. However, the number of studies on gene expression analysis between breeds aimed at understanding how genetic variations affect milk quality is quite limited in sheep as compared to cow. The main difficulty has been, to date, the absence of devices like microarrays. In this paper we have proven that a homologous chip, generated from sheep ESTs, is a valuable tool which can be employed in gene expression analysis. Furthermore, this approach can be easily extended to other species of which genetic sequences are present in public databases [26]. 

## Appendix

**Table 2:** Differentially expressed genes, stage 01.

P value	FC	EST	RefSeq	Gene	UP	DOWN
0.0089536	-4.677	EE874449.1	--	**--**	Gentile	Sarda
0.0399319	-2.705	CD287057.1	--	**--**	Gentile	Sarda
0.00629989	-2.578	EE788795.1	--	**--**	Gentile	Sarda
0.0148422	-2.489	EE873971.1	NM_001083800	**IGLL1**	Gentile	Sarda
0.0107907	-2.339	DY479414.1	--	**--**	Gentile	Sarda
0.00171376	-2.275	EE874479.1	NM_001025317	**RPS8**	Gentile	Sarda
0.00263317	-2.249	EE814758.1	NM_001015592	**PFN1**	Gentile	Sarda
0.00360217	-2.164	EE781281.1	--	**--**	Gentile	Sarda
0.0110063	-2.07	EE866465.1	NM_001033614	**RPS18**	Gentile	Sarda
0.0164299	-2.067	EE873426.1	NM_001105455	**RPL39**	Gentile	Sarda
0.0354098	-2.015	DY497824.1	--	**FTH1**	Gentile	Sarda
0.0306295	-1.918	EE788254.1	--	**--**	Gentile	Sarda
0.0184745	-1.753	EE856480.1	--	**FTH1**	Gentile	Sarda
0.0185062	-1.697	EE824030.1	XM_615127	**ASPHD2**	Gentile	Sarda
0.00474836	-1.682	EE874471.1	NM_001034438	**RPS20**	Gentile	Sarda
0.0434131	-1.632	EE865486.1	NM_001045929	**MPV17**	Gentile	Sarda
0.0250763	-1.588	EE831966.1	--	**--**	Gentile	Sarda
0.019839	-1.582	EE866322.1	XM_615898	**VAV3**	Gentile	Sarda
0.000864936	-1.57	EE848574.1	--	**--**	Gentile	Sarda
0.0424348	-1.568	EE774810.1	XM_001373571	**LOC100021448**	Gentile	Sarda
0.0043326	-1.546	EE866312.1	XM_583460	**RPUSD2**	Gentile	Sarda
0.0440448	-1.522	EE862726.1	XM_001787912	**PLEKHA2**	Gentile	Sarda
0.0263277	-1.52	DY485409.1	--	**--**	Gentile	Sarda
0.04731	-1.514	EE862521.1	NM_001076831	**COL3A1**	Gentile	Sarda
0.0135288	-1.509	EE750744.1	XM_865008	**ERBB2IP**	Gentile	Sarda
0.00245474	-1.492	EE831293.1	--	**--**	Gentile	Sarda
0.0390691	-1.458	EE831369.1	NM_001046226	**FEM1A**	Gentile	Sarda
0.0449235	-1.448	CO202828.1	--	**--**	Gentile	Sarda
0.0107052	-1.445	DY484904.1	--	**--**	Gentile	Sarda
0.0275188	-1.437	EE871094.1	--	**--**	Gentile	Sarda
0.0279427	-1.436	EE864971.1	NM_174590	**PTPN13**	Gentile	Sarda
0.0267974	-1.432	EE792695.1	NM_001101894	**FBXO11**	Gentile	Sarda
0.000833469	-1.419	EE816833.1	NM_001075714	**LOC515452**	Gentile	Sarda
0.000176532	-1.418	EE874214.1	NM_003032	**ST6GAL1**	Gentile	Sarda
0.0471172	-1.404	EE777222.1	NM_001001441	**TNNT3**	Gentile	Sarda
0.0401322	-1.401	EE770300.1	NM_001034333	**SAT1**	Gentile	Sarda
0.00551675	-1.4	DY513982.1	NM_001076011	**ITPKC**	Gentile	Sarda
0.0103614	-1.398	EE802605.1	NM_001078161	**LOC777786**	Gentile	Sarda
0.0244517	-1.389	EE849733.1	--	**--**	Gentile	Sarda
0.00575702	-1.386	EE830948.1	XM_001790594	**LOC100139162**	Gentile	Sarda
0.0251925	-1.377	EE873625.1	NM_001079783	**UBE2E3**	Gentile	Sarda
0.00381358	-1.374	EE816897.1	--	**--**	Gentile	Sarda
0.00653387	-1.373	EE837747.1	NM_001075367	**ALDH2**	Gentile	Sarda
0.0100565	-1.373	EE846386.1	NM_001101154	**ALAS1**	Gentile	Sarda
0.0496638	-1.37	DY500795.1	NM_001035376	**MEOX1**	Gentile	Sarda
0.0090203	-1.367	EE825590.1	XM_001109283	**LOC712430**	Gentile	Sarda
0.015555	-1.361	DY512463.1	NM_001101929	**HK3**	Gentile	Sarda
0.0104062	-1.358	EE807632.1	XM_612129	**ATP2A2**	Gentile	Sarda
0.00419809	-1.357	EE798194.1	XM_001250724	**PDCL**	Gentile	Sarda
0.0129818	-1.347	EE755345.1	XM_865894	**MORC3**	Gentile	Sarda
0.0138409	-1.344	EE795474.1	NM_001105041	**RUSC1**	Gentile	Sarda
0.0268807	-1.342	EE864960.1	NM_001045971	**SPINT2**	Gentile	Sarda
0.0404747	-1.339	EE812525.1	NM_001102149	**MGC159954**	Gentile	Sarda
0.0464112	-1.339	EE869934.1	NM_001100314	**PIK3R4**	Gentile	Sarda
0.027626	-1.338	EE792100.1	--	**--**	Gentile	Sarda
0.00996729	-1.337	EE864412.1	NM_001113764	**TYK2**	Gentile	Sarda
0.0121423	-1.331	EE832631.1	NM_001034743	**RAB5C**	Gentile	Sarda
0.0318396	-1.329	EE831575.1	NM_001046043	**ANGPTL4**	Gentile	Sarda
0.035704	-1.325	EE767566.1	XM_001089198	**beta- galactosidase**	Gentile	Sarda
0.000597784	-1.32	EE857107.1	--	**--**	Gentile	Sarda
0.0323903	-1.319	EE872862.1	NM_001075700	**RAP2C**	Gentile	Sarda
0.0421994	-1.319	EE841857.1	NM_001042682	**RERE**	Gentile	Sarda
0.0208483	-1.309	EE810668.1	NM_001099167	**TMEM149**	Gentile	Sarda
0.0279321	-1.309	EE798509.1	--	**--**	Gentile	Sarda
0.0326586	-1.307	EE842831.1	--	**--**	Gentile	Sarda
0.00133131	-1.304	DY521037.1	XM_001788744	**LOC513508**	Gentile	Sarda
0.0189923	-1.304	EE867731.1	--	**--**	Gentile	Sarda
0.0237616	-1.302	EE816414.1	XM_614378	**SPRYD3**	Gentile	Sarda
0.043391	-1.302	EE858582.1	NM_001077971	**GRSF1**	Gentile	Sarda
0.00992968	-1.301	EE862241.1	NM_175610	**TJP1**	Gentile	Sarda
0.0487321	1.301	EE830752.1	XM_001789168	**LOC100138505**	Sarda	Gentile
0.0153892	1.302	EE770765.1	XM_001787762	**HEXB**	Sarda	Gentile
0.0217868	1.303	EE851241.1	NM_138782	**FCHO2**	Sarda	Gentile
0.0167642	1.303	EE801658.1	NM_001015555	**AUP1**	Sarda	Gentile
0.0430405	1.304	EE831852.1	NM_001102287	**FANCG**	Sarda	Gentile
0.0337551	1.305	EE819075.1	NM_001075848	**RASGRP3**	Sarda	Gentile
0.0152904	1.307	EE759878.1	--	**--**	Sarda	Gentile
0.00792688	1.307	EE869797.1	XM_001789365	**LOC783484**	Sarda	Gentile
0.0305344	1.308	EE839816.1	NM_001038561	**RPUSD3**	Sarda	Gentile
0.00727823	1.309	DY520684.1	XM_001720318	**LOC100129623**	Sarda	Gentile
0.0103058	1.31	EE845343.1	XM_001787123	**CALCA**	Sarda	Gentile
0.000212856	1.311	EE867028.1	NM_001101171	**ABI2**	Sarda	Gentile
0.0198107	1.312	EE782033.1	XM_001256327	**LOC789629**	Sarda	Gentile
0.0405272	1.313	EE783717.1	--	**--**	Sarda	Gentile
0.00239957	1.313	CF117405.1	XM_599530	**LOC521270**	Sarda	Gentile
0.0133575	1.315	EE849952.1	--	**--**	Sarda	Gentile
0.0174528	1.315	EE770796.1	--	**--**	Sarda	Gentile
0.0196397	1.315	EE871964.1	--	**--**	Sarda	Gentile
0.0211185	1.315	EE873622.1	NM_174718	**PNN**	Sarda	Gentile
0.0174924	1.317	EE866027.1	NM_053043	**RBM33**	Sarda	Gentile
0.00224632	1.319	EE833196.1	--	**--**	Sarda	Gentile
0.0077293	1.319	EE746291.1	XR_042970	**LOC532848**	Sarda	Gentile
0.0453195	1.321	EE830242.1	--	**--**	Sarda	Gentile
0.00152261	1.321	EE848826.1	XM_001788615	**ANAPC11**	Sarda	Gentile
0.0371348	1.321	EE861720.1	--	**--**	Sarda	Gentile
0.0124763	1.322	EE840376.1	XR_027670	**LOC539015**	Sarda	Gentile
0.0110695	1.323	EE836611.1	NM_001101080	**ADAMTS1**	Sarda	Gentile
0.00869526	1.324	EE854385.1	--	**--**	Sarda	Gentile
0.0470865	1.325	EE824979.1	NM_001286	**CLCN6**	Sarda	Gentile
0.000591604	1.326	EE834601.1	--	**--**	Sarda	Gentile
0.0164817	1.326	EE872298.1	XM_875686	**CLDND1**	Sarda	Gentile
0.0129429	1.327	EE865545.1	NM_001102062	**WDR75**	Sarda	Gentile
0.00936087	1.33	EE856712.1	XM_001788055	**LOC100138621**	Sarda	Gentile
0.0422128	1.332	EE836140.1	XM_001789542	**LOC100139498**	Sarda	Gentile
0.00099139	1.334	EE865157.1	NM_018509	**LRRC59**	Sarda	Gentile
0.0419962	1.335	EE773093.1	--	**--**	Sarda	Gentile
0.0041349	1.335	EE822639.1	NM_001034368	**ABHD4**	Sarda	Gentile
0.0191105	1.337	EE842849.1	XR_042867	**ZNF134**	Sarda	Gentile
0.0151336	1.338	EE825761.1	XM_001256069	**LOC789273**	Sarda	Gentile
0.0287527	1.338	EE827215.1	NM_001002892	**ST3GAL2**	Sarda	Gentile
0.0106897	1.339	EE861357.1	XM_001255930	**LOC789066**	Sarda	Gentile
0.00547498	1.339	EE796870.1	XM_614279	**LGI2**	Sarda	Gentile
0.0325118	1.341	EE784162.1	--	**--**	Sarda	Gentile
0.0130226	1.342	EE795222.1	--	**IFNAR1E**	Sarda	Gentile
0.0310787	1.342	EE793726.1	--	**--**	Sarda	Gentile
0.0225565	1.343	EE780347.1	--	**--**	Sarda	Gentile
0.0041103	1.343	EE826336.1	NM_001076049	**EFEMP2**	Sarda	Gentile
0.00847258	1.345	EE847787.1	NM_001075670	**SMAP2**	Sarda	Gentile
0.0144838	1.348	EE848020.1	XM_593447	**CCDC61**	Sarda	Gentile
0.0251965	1.348	DY491137.1	--	**--**	Sarda	Gentile
0.01472	1.35	EE747969.1	--	**--**	Sarda	Gentile
0.0230545	1.355	EE872727.1	NM_001078102	**DNAJC24**	Sarda	Gentile
0.0340345	1.357	EE797130.1	NM_020772	**NUFIP2**	Sarda	Gentile
0.0400043	1.357	EE782700.1	--	**--**	Sarda	Gentile
0.0199472	1.358	EE852157.1	NM_172127	**CAMK2D**	Sarda	Gentile
0.0284178	1.36	EE837214.1	NM_001098003	**TMCO3**	Sarda	Gentile
0.0152216	1.36	EE844290.1	NM_001102498	**NKAPL**	Sarda	Gentile
0.000664005	1.36	EE816910.1	--	**--**	Sarda	Gentile
0.0129656	1.365	EE813986.1	XM_584123	**LOC538993**	Sarda	Gentile
0.00239596	1.368	EE833638.1	--	**--**	Sarda	Gentile
0.00473005	1.371	EE776285.1	XM_600379	**AHDC1**	Sarda	Gentile
0.0313393	1.372	EE837130.1	NM_001025345	**MCM7**	Sarda	Gentile
0.00257839	1.373	DY522523.1	NM_001102074	**QSOX1**	Sarda	Gentile
0.0308861	1.373	EE868991.1	NM_001034633	**SLC3A1**	Sarda	Gentile
0.0407681	1.375	EE746824.1	XM_865771	**RUNX1**	Sarda	Gentile
0.0393277	1.379	EE794485.1	--	**--**	Sarda	Gentile
0.0307812	1.38	EE815257.1	NM_001046011	**CD37**	Sarda	Gentile
0.0184587	1.384	EE816699.1	NM_001083429	**PANK4**	Sarda	Gentile
0.000606351	1.384	EE830551.1	NM_001037607	**ARFRP1**	Sarda	Gentile
0.0424528	1.385	EE753287.1	--	**--**	Sarda	Gentile
0.0213158	1.385	EE826333.1	XM_590179	**DENND2A**	Sarda	Gentile
0.0264585	1.386	EE851940.1	NM_012319	**SLC39A6**	Sarda	Gentile
0.00647171	1.387	EE790636.1	NM_001045969	**ALDH7A1**	Sarda	Gentile
0.0226806	1.388	CN824197.1	NM_053064	**GNG2**	Sarda	Gentile
0.0387653	1.388	EE813255.1	NM_030935	**TSC22D4**	Sarda	Gentile
0.00174915	1.393	EE791965.1	NM_001046497	**MAT1A**	Sarda	Gentile
0.0264875	1.397	DY490978.1	--	**NDUFS2**	Sarda	Gentile
0.00295738	1.398	EE829627.1	NM_001077854	**MS4A1**	Sarda	Gentile
0.0224081	1.399	EE854207.1	NM_001077104	**KRTAP3-1**	Sarda	Gentile
0.0470249	1.402	EE858765.1	NM_001083703	**RNF185**	Sarda	Gentile
0.00371868	1.402	EE832497.1	NM_001078041	**PLD3**	Sarda	Gentile
0.0216581	1.402	EE866310.1	NM_001099072	**UNC45A**	Sarda	Gentile
0.010359	1.403	EE808144.1	XM_001250150	**TAF2**	Sarda	Gentile
0.0317868	1.407	EE833224.1	--	**CDH5**	Sarda	Gentile
0.00506313	1.408	EE794195.1	--	**--**	Sarda	Gentile
0.0323786	1.408	DY478310.1	NM_000088	**COL1A1**	Sarda	Gentile
0.00625366	1.41	EE825745.1	NM_001102546	**PDE4B**	Sarda	Gentile
0.0396624	1.412	EE764887.1	--	**--**	Sarda	Gentile
0.0240626	1.413	EE818092.1	NM_001102238	**METTL3**	Sarda	Gentile
0.000452341	1.416	EE843072.1	NM_178140	**PDZD2**	Sarda	Gentile
0.00772543	1.418	EE856741.1	NM_001075176	**RPA1**	Sarda	Gentile
0.0055955	1.421	EE823634.1	--	**--**	Sarda	Gentile
0.0140254	1.422	EE864563.1	NM_001034339	**IL11RA**	Sarda	Gentile
4.02e-05	1.424	DY480261.1	--	**--**	Sarda	Gentile
0.0444903	1.428	EE816651.1	NM_001083414	**WWP2**	Sarda	Gentile
0.000877121	1.432	EE870673.1	--	**--**	Sarda	Gentile
0.0154273	1.442	EE766357.1	XM_585246	**KIF11**	Sarda	Gentile
0.00750741	1.446	EE825953.1	XM_865238	**EXOC2**	Sarda	Gentile
0.00222257	1.446	EE788062.1	NM_019863	**F8**	Sarda	Gentile
0.00743356	1.446	EE747821.1	--	**--**	Sarda	Gentile
0.0372742	1.447	EE871308.1	--	**--**	Sarda	Gentile
0.015438	1.448	EE851254.1	--	**--**	Sarda	Gentile
0.0081182	1.451	DY520937.1	NM_001102035	**CERCAM**	Sarda	Gentile
0.00371839	1.458	EE873738.1	NM_001035283	**TALDO1**	Sarda	Gentile
0.00612951	1.458	EE765691.1	--	**GALN**	Sarda	Gentile
0.0211205	1.46	EE780060.1	NM_001012281	**CFLAR **	Sarda	Gentile
0.0149215	1.461	EE821548.1	NM_001046390	**TES**	Sarda	Gentile
0.0337644	1.464	BG874259.1	--	**--**	Sarda	Gentile
0.0347533	1.466	DY514734.1	XM_589271	**FBLN2**	Sarda	Gentile
0.00652737	1.469	EE783524.1	--	**--**	Sarda	Gentile
0.0121699	1.469	EE828474.1	XM_870386	**SH3BP5**	Sarda	Gentile
0.00171057	1.477	DY504539.1	--	**--**	Sarda	Gentile
0.00492899	1.478	EE856049.1	XM_865072	**PIGT**	Sarda	Gentile
0.00154659	1.479	EE871601.1	--	**--**	Sarda	Gentile
0.0138137	1.48	EE831423.1	XM_001788882	**LOC789539**	Sarda	Gentile
0.00952563	1.481	EE829269.1	NM_001014865	**DRG2**	Sarda	Gentile
0.00808543	1.489	EE818202.1	XM_602855	**GALNT7**	Sarda	Gentile
0.0212484	1.491	DY485302.1	NM_001024824	**RIPK5**	Sarda	Gentile
0.0204847	1.498	EE871638.1	XM_870378	**CAPN7**	Sarda	Gentile
0.0185014	1.511	DY480855.1	XM_001787789	**LOC534471**	Sarda	Gentile
0.0161177	1.514	EE748326.1	NM_001046194	**CDCA2**	Sarda	Gentile
0.00825915	1.514	DY496078.1	XM_001789157	**LOC508459**	Sarda	Gentile
0.0193849	1.517	EE841331.1	NM_001039591	**USP9X**	Sarda	Gentile
0.00544002	1.54	EE767854.1	NM_001076330	**PRSS16**	Sarda	Gentile
0.00694391	1.541	EE820835.1	NM_001045866	**BRD2**	Sarda	Gentile
0.048183	1.544	EE760222.1	XM_001252101	**LOC784704**	Sarda	Gentile
0.00585242	1.551	DY491388.1	XM_596546	**SH3PXD2B**	Sarda	Gentile
0.0242248	1.554	EE749164.1	NM_174676	**RASA3**	Sarda	Gentile
0.0179786	1.561	EE750374.1	--	**--**	Sarda	Gentile
0.0452829	1.564	EE857270.1	XM_596854	**PLEK**	Sarda	Gentile
0.036256	1.566	EE798015.1	--	**--**	Sarda	Gentile
0.00057357	1.579	EE856496.1	NM_001046252	**LITAF**	Sarda	Gentile
0.0123283	1.599	EE825369.1	NM_001081602	**STK38**	Sarda	Gentile
0.00833088	1.603	EE812445.1	XM_001254977	**LRRCC1**	Sarda	Gentile
0.0428815	1.612	EE826810.1	NM_001035012	**RIPK1**	Sarda	Gentile
0.00179087	1.642	DY500867.1	XM_001254158	**ZNF318**	Sarda	Gentile
0.00631294	1.682	EE849102.1	--	**--**	Sarda	Gentile
7.62e-05	1.877	EE799074.1	NM_174703	**TNXB**	Sarda	Gentile
0.0144325	1.931	EE791703.1	--	**--**	Sarda	Gentile
0.0124589	2.018	EE874444.1	--	**CSN2**	Sarda	Gentile
0.0468452	2.039	EE816347.1	NM_001075485	**LYSMD2**	Sarda	Gentile
0.0228427	2.078	EE874341.1	--	**LOC443383**	Sarda	Gentile
0.0176061	2.197	EE874443.1	--	**CSN3**	Sarda	Gentile

**Table 3:** Differentially expressed genes, stage 02.

pvalue	FC	EST	RefSeq	Gene	UP	DOWN
0.0262055	-1.467	EE855884.1	NM_001038689	****C5H12orf45****	Gentile	Sarda
0.0440456	-1.391	EE804883.1	NM_172216	****CAMKK2****	Gentile	Sarda
0.0295858	-1.37	EE752417.1	NM_174438	****PROS1****	Gentile	Sarda
0.00912544	-1.344	EE805588.1	NM_001105615	****CENPP****	Gentile	Sarda
0.0356366	-1.341	CF117455.1	XM_001254445	****FBXO48****	Gentile	Sarda
0.0153359	-1.332	EE831205.1	NM_001046346	****WDR1****	Gentile	Sarda
0.0167085	-1.304	EE872615.1	NM_001099859	****EIF4G2****	Gentile	Sarda
0.00236899	-1.302	EE803274.1	NM_001102100	****LENG8****	Gentile	Sarda
0.0220397	1.303	EE794531.1	NM_001083487	****DAGLB****	Sarda	Gentile
0.0117502	1.305	CD288635.1	NM_001083793	****SMAGP****	Sarda	Gentile
0.00915309	1.308	EE811805.1	--	****--****	Sarda	Gentile
0.0480977	1.314	DY516851.1	XR_028016	****LOC539596****	Sarda	Gentile
0.00597485	1.316	EE869114.1	--	****--****	Sarda	Gentile
0.0159031	1.316	EE749850.1	XM_001249987	****EEF1A1****	Sarda	Gentile
0.0483209	1.32	EE810406.1	NM_001045938	****AP1B1****	Sarda	Gentile
0.0164487	1.322	EE840745.1	NM_194442	****LBR****	Sarda	Gentile
0.0194088	1.323	EE752798.1	NM_001083462	****SEC22A****	Sarda	Gentile
0.0205422	1.324	DY514991.1	XM_001251051	****LOC782414****	Sarda	Gentile
0.00825489	1.325	EE872139.1	XR_027898	****LOC784355****	Sarda	Gentile
0.000861353	1.327	EE858050.1	XM_580387	****PRKD3****	Sarda	Gentile
0.0386497	1.33	EE788388.1	--	****--****	Sarda	Gentile
0.000485662	1.335	EE844249.1	NM_001075142	****IL4R****	Sarda	Gentile
0.00842772	1.336	EE816344.1	--	****--****	Sarda	Gentile
0.0290668	1.338	EE874443.1	--	****CSN3****	Sarda	Gentile
0.032805	1.342	EE834071.1	XM_583748	****TBX21****	Sarda	Gentile
0.00363329	1.359	EE860008.1	XM_613386	****ZNF532****	Sarda	Gentile
0.000451745	1.361	DY520339.1	NM_174520	****COL1A2****	Sarda	Gentile
0.028263	1.362	DY480878.1	--	****--****	Sarda	Gentile
0.046296	1.392	EE820416.1	--	****--****	Sarda	Gentile
0.00529847	1.405	DY520664.1	NM_001075474	****MRPS16****	Sarda	Gentile
0.0195754	1.417	CF117857.1	NM_001098860	****RAMP2****	Sarda	Gentile
0.00072133	1.456	CD287057.1	--	****--****	Sarda	Gentile
0.00198483	1.465	DY479000.1	--	****--****	Sarda	Gentile
0.022287	1.47	EE801908.1	NM_001046214	****ACBD4****	Sarda	Gentile
0.0105584	1.484	EE815399.1	XM_589331	****TGOLN2****	Sarda	Gentile
0.00657655	1.518	DY497824.1	--	****FTH1****	Sarda	Gentile
